# Crystal structure of (*E*)-*N*-[(*E*)-3-(4-meth­oxy­phen­yl)allyl­idene]naphthalen-1-amine

**DOI:** 10.1107/S1600536814022521

**Published:** 2014-10-24

**Authors:** Jae Kyun Lee, Joo Hwan Cha, Yong Seo Cho, Sun-Joon Min, Joon Kyun Lee

**Affiliations:** a Center for Neuro-Medicine, Brain Science Institute, Korea Institute of Science & Technology, Hwarangro 14-gil, Seongbuk-gu, Seoul 136-791, Republic of Korea; b Advanced Analysis Center, Korea Institute of Science & Technology, Hwarangro 14-gil, Seongbuk-gu, Seoul 136-791, Republic of Korea; c Micro/Nano Scale Manufacturing R&BD Group, Korea Institute of Industrial Technology, 143 Hanggaulro, Sangnok-gu, Ansan-si, Gyeonggi-do 426-910, Republic of Korea

**Keywords:** crystal structure, naphthalene derivative, π–π inter­actions

## Abstract

In the title compound, C_20_H_17_NO, the dihedral angle between the mean planes of the 4-meth­oxy­phenyl ring and the naphthalene ring is 69.50 (7)°. The meth­oxy group is almost coplanar with the benzene ring to which it is connected [Cb—Cb—Om—Cm torsion angle of −7.9 (2)°; b = benzene and m = meth­oxy] and the imine group displays a C—C—N=C torsion angle is −57.2 (2)°. The imine (C=N) group has an *E* conformation. In the crystal, weak π–π inter­actions between the benzene rings [centroid–centroid distance = 3.7781 (10) Å] are observed.

## Related literature   

For the uses of naphthalene derivatives in various scientific fields, see: Ohta *et al.* (2005 ▶) and references therein. For background information and related crystal structures studied recently by our group, see: Lee *et al.* (2013 ▶); Nam *et al.* (2013 ▶).
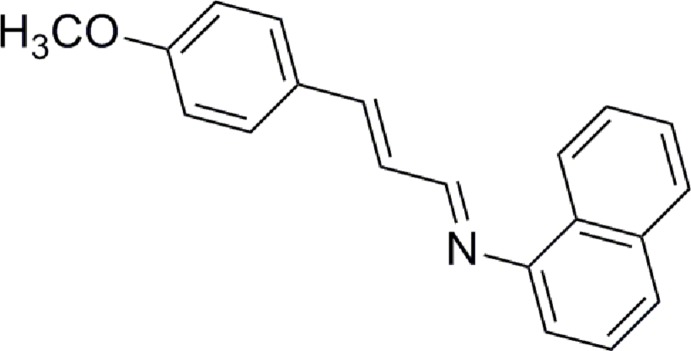


## Experimental   

### Crystal data  

C_20_H_17_NO
*M* *_r_* = 287.36
Triclinic, 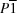

*a* = 7.8278 (8) Å
*b* = 9.8931 (10) Å
*c* = 11.3929 (13) Å
α = 69.307 (3)°
β = 73.561 (3)°
γ = 81.375 (3)°
*V* = 790.40 (15) Å^3^
*Z* = 2
Mo *K*α radiation
μ = 0.07 mm^−1^
*T* = 296 K
0.30 × 0.20 × 0.20 mm

### Data collection  

Rigaku R-AXIS RAPID diffractometer
Absorption correction: multi-scan (*ABSCOR*; Rigaku, 1995 ▶) *T*_min_ = 0.701, *T*_max_ = 0.985
7842 measured reflections
3584 independent reflections
1680 reflections with *F* ^2^ > 2σ(*F* ^2^)
*R*_int_ = 0.023

### Refinement  

*R*[*F* ^2^ > 2σ(*F* ^2^)] = 0.038
*wR*(*F* ^2^) = 0.119
*S* = 0.99
3584 reflections
212 parameters
H atoms treated by a mixture of independent and constrained refinement
Δρ_max_ = 0.15 e Å^−3^
Δρ_min_ = −0.23 e Å^−3^

### 

Data collection: *RAPID-AUTO* (Rigaku, 2006 ▶); cell refinement: *RAPID-AUTO*; data reduction: *RAPID-AUTO*; program(s) used to solve structure: *Il Milione* (Burla *et al.*, 2007 ▶); program(s) used to refine structure: *SHELXL97* (Sheldrick, 2008 ▶); molecular graphics: *CrystalStructure* (Rigaku, 2010 ▶); software used to prepare material for publication: *CrystalStructure*.

## Supplementary Material

Crystal structure: contains datablock(s) General, I. DOI: 10.1107/S1600536814022521/ff2132sup1.cif

Structure factors: contains datablock(s) I. DOI: 10.1107/S1600536814022521/ff2132Isup2.hkl

Supporting information file. DOI: 10.1107/S1600536814022521/ff2132Isup3.cml

**Fig. 1.**
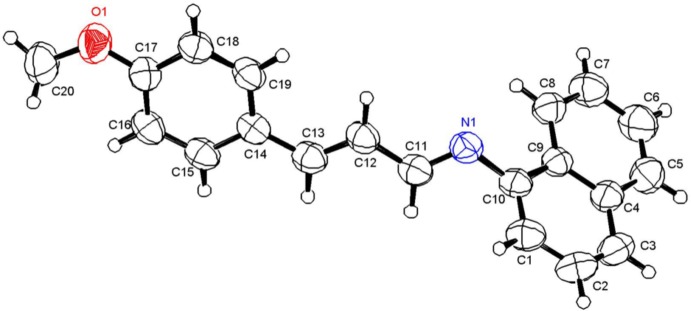
The molecular structure of the title compound showing the atomic numbering and 50% probability displacement ellipsoids.

. DOI: 10.1107/S1600536814022521/ff2132fig1.tif
The mol­ecular structure of the title compound showing the atomic numbering and 50% probability displacement ellipsoids.

CCDC reference: 1029010

Additional supporting information:  crystallographic information; 3D view; checkCIF report

## supplementary crystallographic information

### S1. Experimental

To a solution of 1-naphthylamine (2.0 mmol) in anhydrous ethanol (40 ml) was treated with equimolar quantity of 4-methoxycinnamaldehyde. The mixture was refluxed for 2 days, and the progress of reaction was monitored by TLC. After completion of reaction, the solvent was removed under reduced pressure. The residue was purified by flash column chromatography to afford the title compound as a yellow solid in yield 92%. Single crystals suitable for X-ray diffraction were prepared by slow evaporation of a solution of the title compound in cosolvent(ethyl acetate 1: hexane 3) at room temperature.

### S2. Refinement

All hydrogen atoms were positioned geometrically and refined using a riding model with C—H = 0.93–0.99 Å and *U*iso(H) = 1.2 or 1.5 *U*eq(C).

### Crystal data

**Table:**

C_20_H_17_NO	*Z* = 2
*M**_r_* = 287.36	*F*(000) = 304.00
Triclinic, *P*1	*D*_x_ = 1.207 Mg m^−^^3^
Hall symbol: -P 1	Mo *K*α radiation, λ = 0.71075 Å
*a* = 7.8278 (8) Å	Cell parameters from 4416 reflections
*b* = 9.8931 (10) Å	θ = 3.2–27.5°
*c* = 11.3929 (13) Å	µ = 0.07 mm^−^^1^
α = 69.307 (3)°	*T* = 296 K
β = 73.561 (3)°	Block, yellow
γ = 81.375 (3)°	0.30 × 0.20 × 0.20 mm
*V* = 790.40 (15) Å^3^	

### Data collection

**Table:**

Rigaku R-AXIS RAPID diffractometer	1680 reflections with *F*^2^ > 2σ(*F*^2^)
Detector resolution: 10.000 pixels mm^-1^	*R*_int_ = 0.023
ω scans	θ_max_ = 27.5°
Absorption correction: multi-scan (*ABSCOR*; Rigaku, 1995)	*h* = −10→10
*T*_min_ = 0.701, *T*_max_ = 0.985	*k* = −12→12
7842 measured reflections	*l* = −14→14
3584 independent reflections	

### Refinement

**Table:**

Refinement on *F*^2^	Secondary atom site location: difference Fourier map
*R*[*F*^2^ > 2σ(*F*^2^)] = 0.038	Hydrogen site location: inferred from neighbouring sites
*wR*(*F*^2^) = 0.119	H atoms treated by a mixture of independent and constrained refinement
*S* = 0.99	*w* = 1/[σ^2^(*F*_o_^2^) + (0.059*P*)^2^] where *P* = (*F*_o_^2^ + 2*F*_c_^2^)/3
3584 reflections	(Δ/σ)_max_ < 0.001
212 parameters	Δρ_max_ = 0.15 e Å^−^^3^
0 restraints	Δρ_min_ = −0.23 e Å^−^^3^
Primary atom site location: structure-invariant direct methods	

### Special details

**Table:**

Geometry. All e.s.d.'s (except the e.s.d. in the dihedral angle between two l.s. planes) are estimated using the full covariance matrix. The cell e.s.d.'s are taken into account individually in the estimation of e.s.d.'s in distances, angles and torsion angles; correlations between e.s.d.'s in cell parameters are only used when they are defined by crystal symmetry. An approximate (isotropic) treatment of cell e.s.d.'s is used for estimating e.s.d.'s involving l.s. planes.
Refinement. Refinement was performed using all reflections. The weighted *R*-factor (*wR*) and goodness of fit (*S*) are based on *F*^2^. *R*-factor (gt) are based on *F*. The threshold expression of *F*^2^ > 2.0 σ(*F*^2^) is used only for calculating *R*-factor (gt).

### Fractional atomic coordinates and isotropic or equivalent isotropic displacement parameters (Å^2^)

**Table:**

	*x*	*y*	*z*	*U*_iso_*/*U*_eq_	
O1	0.89882 (16)	−0.21021 (11)	0.81880 (12)	0.0803 (4)	
N1	0.41441 (15)	0.54539 (14)	0.29989 (12)	0.0598 (4)	
C1	0.4683 (2)	0.68327 (18)	0.06861 (16)	0.0680 (5)	
C2	0.4038 (3)	0.77286 (19)	−0.03750 (16)	0.0747 (5)	
C3	0.2272 (3)	0.80938 (18)	−0.02523 (16)	0.0705 (5)	
C4	0.10408 (19)	0.75954 (15)	0.09645 (14)	0.0564 (4)	
C5	−0.0813 (3)	0.79622 (18)	0.11416 (17)	0.0725 (5)	
C6	−0.1958 (2)	0.7484 (2)	0.23285 (19)	0.0782 (5)	
C7	−0.1328 (2)	0.66267 (18)	0.34010 (17)	0.0721 (5)	
C8	0.04414 (19)	0.62462 (16)	0.32731 (15)	0.0599 (4)	
C9	0.16784 (17)	0.67163 (14)	0.20559 (13)	0.0491 (4)	
C10	0.35441 (18)	0.63154 (15)	0.18883 (14)	0.0537 (4)	
C11	0.50541 (19)	0.42769 (17)	0.29784 (16)	0.0599 (4)	
C12	0.57334 (18)	0.33343 (17)	0.40571 (17)	0.0596 (4)	
C13	0.64317 (19)	0.19989 (18)	0.41200 (17)	0.0614 (4)	
C14	0.70825 (16)	0.09209 (15)	0.51729 (14)	0.0536 (4)	
C15	0.76728 (18)	−0.04619 (16)	0.51217 (16)	0.0608 (4)	
C16	0.83062 (18)	−0.15070 (16)	0.61020 (16)	0.0613 (4)	
C17	0.83684 (18)	−0.11806 (15)	0.71615 (16)	0.0577 (4)	
C18	0.7792 (2)	0.01956 (16)	0.72397 (16)	0.0651 (5)	
C19	0.71601 (18)	0.12164 (16)	0.62642 (15)	0.0609 (5)	
C20	0.9415 (3)	−0.35674 (17)	0.82635 (19)	0.0885 (6)	
H1	0.5898	0.6584	0.0576	0.0816*	
H2	0.4835	0.8080	−0.1179	0.0897*	
H3	0.1868	0.8676	−0.0974	0.0847*	
H5	−0.1252	0.8539	0.0435	0.0870*	
H6	−0.3172	0.7732	0.2426	0.0938*	
H7	−0.2123	0.6312	0.4211	0.0865*	
H8	0.0843	0.5669	0.3997	0.0718*	
H15	0.7639	−0.0689	0.4403	0.0730*	
H16	0.8687	−0.2423	0.6044	0.0735*	
H18	0.7837	0.0419	0.7957	0.0781*	
H19	0.6774	0.2129	0.6331	0.0730*	
H20A	0.8377	−0.3992	0.8283	0.1062*	
H20B	1.0349	−0.3619	0.7521	0.1062*	
H20C	0.9808	−0.4086	0.9037	0.1062*	
H13	0.649 (2)	0.1693 (17)	0.3375 (17)	0.079 (5)*	
H12	0.5628 (18)	0.3682 (16)	0.4790 (16)	0.068 (5)*	
H11	0.5287 (18)	0.3968 (15)	0.2201 (15)	0.066 (5)*	

### Atomic displacement parameters (Å^2^)

**Table:**

	*U*^11^	*U*^22^	*U*^33^	*U*^12^	*U*^13^	*U*^23^
O1	0.1091 (9)	0.0578 (7)	0.0842 (9)	0.0041 (6)	−0.0426 (7)	−0.0244 (7)
N1	0.0581 (7)	0.0672 (8)	0.0580 (8)	0.0028 (6)	−0.0176 (6)	−0.0249 (7)
C1	0.0576 (9)	0.0868 (12)	0.0616 (11)	−0.0092 (8)	−0.0069 (8)	−0.0310 (9)
C2	0.0780 (12)	0.0922 (13)	0.0484 (10)	−0.0202 (9)	−0.0031 (9)	−0.0199 (10)
C3	0.0879 (12)	0.0742 (11)	0.0495 (10)	−0.0087 (9)	−0.0203 (9)	−0.0161 (8)
C4	0.0674 (10)	0.0550 (9)	0.0543 (9)	−0.0044 (7)	−0.0197 (8)	−0.0227 (8)
C5	0.0743 (11)	0.0790 (12)	0.0742 (12)	0.0069 (9)	−0.0337 (10)	−0.0295 (10)
C6	0.0582 (10)	0.1012 (14)	0.0835 (14)	0.0039 (9)	−0.0213 (10)	−0.0409 (12)
C7	0.0567 (10)	0.0882 (12)	0.0686 (12)	−0.0073 (8)	−0.0049 (9)	−0.0293 (10)
C8	0.0614 (9)	0.0612 (10)	0.0548 (10)	−0.0055 (7)	−0.0115 (8)	−0.0180 (8)
C9	0.0563 (8)	0.0464 (8)	0.0489 (9)	−0.0057 (6)	−0.0129 (7)	−0.0197 (7)
C10	0.0570 (9)	0.0594 (9)	0.0524 (9)	−0.0044 (7)	−0.0138 (7)	−0.0268 (8)
C11	0.0543 (9)	0.0688 (10)	0.0611 (10)	−0.0011 (7)	−0.0127 (8)	−0.0289 (9)
C12	0.0508 (8)	0.0699 (11)	0.0643 (11)	0.0021 (7)	−0.0156 (8)	−0.0305 (9)
C13	0.0560 (9)	0.0712 (11)	0.0645 (11)	−0.0021 (7)	−0.0151 (8)	−0.0319 (9)
C14	0.0466 (8)	0.0581 (9)	0.0621 (10)	−0.0033 (6)	−0.0117 (7)	−0.0283 (8)
C15	0.0606 (9)	0.0666 (10)	0.0675 (11)	−0.0029 (7)	−0.0163 (8)	−0.0365 (9)
C16	0.0629 (9)	0.0546 (9)	0.0753 (11)	−0.0013 (7)	−0.0174 (8)	−0.0328 (9)
C17	0.0581 (9)	0.0538 (9)	0.0645 (10)	−0.0058 (7)	−0.0176 (8)	−0.0206 (8)
C18	0.0795 (11)	0.0617 (10)	0.0659 (11)	0.0001 (8)	−0.0235 (9)	−0.0325 (9)
C19	0.0635 (9)	0.0582 (9)	0.0705 (11)	0.0037 (7)	−0.0189 (8)	−0.0336 (9)
C20	0.1086 (14)	0.0560 (11)	0.0975 (15)	0.0034 (9)	−0.0321 (12)	−0.0192 (10)

### Geometric parameters (Å, º)

**Table:**

O1—C17	1.367 (2)	C16—C17	1.370 (3)
O1—C20	1.415 (2)	C17—C18	1.394 (3)
N1—C10	1.415 (2)	C18—C19	1.367 (3)
N1—C11	1.275 (2)	C1—H1	0.930
C1—C2	1.399 (3)	C2—H2	0.930
C1—C10	1.3723 (19)	C3—H3	0.930
C2—C3	1.356 (3)	C5—H5	0.930
C3—C4	1.413 (2)	C6—H6	0.930
C4—C5	1.416 (3)	C7—H7	0.930
C4—C9	1.417 (2)	C8—H8	0.930
C5—C6	1.358 (3)	C11—H11	0.996 (19)
C6—C7	1.393 (3)	C12—H12	0.99 (2)
C7—C8	1.360 (2)	C13—H13	0.98 (2)
C8—C9	1.4129 (18)	C15—H15	0.930
C9—C10	1.4313 (19)	C16—H16	0.930
C11—C12	1.440 (3)	C18—H18	0.930
C12—C13	1.337 (3)	C19—H19	0.930
C13—C14	1.454 (3)	C20—H20A	0.960
C14—C15	1.393 (3)	C20—H20B	0.960
C14—C19	1.393 (3)	C20—H20C	0.960
C15—C16	1.380 (3)		
			
O1···C19	3.5931 (18)	C16···H2^vi^	3.4384
N1···C8	2.8446 (19)	C17···H2^vi^	2.9036
N1···C13	3.591 (2)	C17···H15^v^	3.5517
C1···C4	2.795 (2)	C18···H2^vi^	3.0680
C1···C11	2.973 (2)	C18···H15^v^	3.5526
C2···C9	2.7901 (19)	C19···H6^viii^	3.2467
C3···C10	2.802 (3)	C19···H8^iii^	3.5445
C4···C7	2.793 (2)	C20···H1^vi^	3.2542
C5···C8	2.771 (3)	C20···H3^i^	3.5996
C6···C9	2.792 (2)	C20···H20C^xiv^	3.2874
C9···C11	3.432 (2)	H1···O1^ix^	3.1078
C12···C19	3.021 (3)	H1···C5^vii^	3.3934
C14···C17	2.793 (2)	H1···C6^vii^	3.3460
C15···C18	2.740 (3)	H1···C20^ix^	3.2542
C16···C19	2.762 (3)	H1···H5^vii^	3.1004
C16···C20	2.847 (3)	H1···H6^vii^	3.0108
O1···C3^i^	3.575 (3)	H1···H20A^ix^	2.9604
O1···C5^i^	3.436 (3)	H1···H20C^ix^	3.1705
C3···O1^ii^	3.575 (3)	H2···O1^ix^	3.1163
C5···O1^ii^	3.436 (3)	H2···C11^x^	3.5870
C9···C18^iii^	3.529 (3)	H2···C16^ix^	3.4384
C9···C20^iv^	3.514 (3)	H2···C17^ix^	2.9036
C13···C15^iv^	3.514 (3)	H2···C18^ix^	3.0680
C14···C16^v^	3.5413 (19)	H2···H18^ix^	3.2497
C15···C13^iv^	3.514 (3)	H2···H20A^ix^	3.2383
C16···C14^v^	3.5413 (19)	H2···H13^x^	2.8988
C18···C9^iii^	3.529 (3)	H2···H11^x^	2.6953
C20···C9^iv^	3.514 (3)	H3···O1^ii^	2.9743
O1···H16	2.6497	H3···C5^xi^	3.2551
O1···H18	2.4698	H3···C6^xi^	3.5624
N1···H1	2.6251	H3···C20^ii^	3.5996
N1···H8	2.5217	H3···H5^xi^	2.9716
N1···H12	2.605 (16)	H3···H6^xi^	3.5192
C1···H3	3.2362	H3···H20C^ii^	3.3696
C1···H11	2.799 (13)	H3···H13^x^	2.8004
C3···H1	3.2290	H3···H11^x^	3.5828
C3···H5	2.6593	H5···O1^ii^	2.7982
C4···H2	3.2415	H5···C3^xi^	3.2536
C4···H6	3.2532	H5···C4^xi^	3.5955
C4···H8	3.2748	H5···H1^xii^	3.1004
C5···H3	2.6625	H5···H3^xi^	2.9716
C5···H7	3.2219	H5···H5^xi^	3.4288
C6···H8	3.2269	H5···H18^ii^	2.9949
C7···H5	3.2253	H5···H20C^ii^	3.3902
C8···H6	3.2234	H6···N1^xii^	3.0857
C9···H1	3.2629	H6···C1^xii^	3.3336
C9···H3	3.2803	H6···C10^xii^	3.3944
C9···H5	3.2749	H6···C19^viii^	3.2467
C9···H7	3.2508	H6···H1^xii^	3.0108
C10···H2	3.2399	H6···H3^xi^	3.5192
C10···H8	2.6639	H6···H15^xiii^	3.3938
C10···H11	2.474 (14)	H6···H19^viii^	2.7811
C11···H1	2.8555	H6···H12^viii^	3.1520
C11···H8	3.4223	H7···N1^viii^	3.1177
C11···H13	2.573 (16)	H7···C8^viii^	3.5163
C12···H19	2.7411	H7···C11^viii^	3.2841
C13···H15	2.6229	H7···C12^viii^	3.0068
C13···H19	2.6553	H7···H8^viii^	2.5990
C13···H11	2.637 (15)	H7···H15^xiii^	3.0266
C14···H16	3.2628	H7···H16^xiii^	3.0273
C14···H18	3.2431	H7···H12^xii^	3.1315
C14···H12	2.731 (15)	H7···H12^viii^	2.6565
C15···H19	3.2194	H8···C7^viii^	3.1161
C15···H13	2.608 (16)	H8···C8^viii^	2.9850
C16···H18	3.2298	H8···C19^iii^	3.5445
C16···H20A	2.8179	H8···H7^viii^	2.5990
C16···H20B	2.7583	H8···H8^viii^	2.3227
C17···H15	3.2076	H8···H16^xiii^	3.4321
C17···H19	3.2288	H8···H16^iv^	3.1918
C17···H20A	2.6182	H8···H19^iii^	2.9362
C17···H20B	2.6321	H8···H20A^iv^	3.4229
C17···H20C	3.1856	H8···H20B^iv^	3.4544
C18···H16	3.2356	H15···C6^xv^	3.3733
C19···H15	3.2172	H15···C7^xv^	3.1604
C19···H13	3.34 (2)	H15···C13^iv^	3.3659
C19···H12	2.753 (14)	H15···C17^v^	3.5517
C20···H16	2.5691	H15···C18^v^	3.5526
H1···H2	2.3150	H15···H6^xv^	3.3938
H1···H11	2.6153	H15···H7^xv^	3.0266
H2···H3	2.2748	H15···H13^iv^	3.5238
H3···H5	2.5070	H16···C7^xv^	3.4602
H5···H6	2.2784	H16···C8^iv^	3.5808
H6···H7	2.3162	H16···C11^iv^	3.3910
H7···H8	2.2802	H16···C14^v^	3.5385
H15···H16	2.2998	H16···H7^xv^	3.0273
H15···H13	2.3948	H16···H8^xv^	3.4321
H16···H20A	2.4372	H16···H8^iv^	3.1918
H16···H20B	2.3010	H16···H11^iv^	3.4288
H16···H20C	3.5218	H18···C3^iii^	3.4003
H18···H19	2.2854	H18···C4^iii^	2.9952
H19···H12	2.1952	H18···C5^iii^	3.5603
H13···H12	2.86 (3)	H18···C8^iii^	3.4145
H13···H11	2.39 (2)	H18···C9^iii^	2.9091
H12···H11	2.95 (3)	H18···C10^iii^	3.2988
O1···H1^vi^	3.1078	H18···H2^vi^	3.2497
O1···H2^vi^	3.1163	H18···H5^i^	2.9949
O1···H3^i^	2.9743	H19···N1^iii^	2.6991
O1···H5^i^	2.7982	H19···C8^iii^	3.1087
O1···H13^v^	3.492 (14)	H19···C9^iii^	3.0520
N1···H6^vii^	3.0857	H19···C10^iii^	2.8908
N1···H7^viii^	3.1177	H19···H6^viii^	2.7811
N1···H19^iii^	2.6991	H19···H8^iii^	2.9362
N1···H20A^iv^	3.4788	H20A···N1^iv^	3.4788
N1···H12^iii^	2.99 (2)	H20A···C1^vi^	3.5932
C1···H6^vii^	3.3336	H20A···C4^iv^	3.3507
C1···H20A^ix^	3.5932	H20A···C8^iv^	3.1971
C2···H13^x^	3.40 (2)	H20A···C9^iv^	2.8631
C2···H11^x^	3.002 (19)	H20A···C10^iv^	3.0175
C3···H5^xi^	3.2536	H20A···C11^iv^	3.4725
C3···H18^iii^	3.4003	H20A···H1^vi^	2.9604
C3···H20C^ii^	3.5201	H20A···H2^vi^	3.2383
C3···H13^x^	3.350 (19)	H20A···H8^iv^	3.4229
C3···H11^x^	3.518 (17)	H20A···H20C^xiv^	3.5351
C4···H5^xi^	3.5955	H20A···H11^iv^	3.0661
C4···H18^iii^	2.9952	H20B···C7^iv^	3.4084
C4···H20A^iv^	3.3507	H20B···C8^iv^	3.2280
C4···H20C^ii^	3.5750	H20B···C9^iv^	3.4819
C5···H1^xii^	3.3934	H20B···C11^v^	3.4783
C5···H3^xi^	3.2551	H20B···C12^v^	3.0819
C5···H18^iii^	3.5603	H20B···C13^v^	2.9736
C5···H20C^ii^	3.5450	H20B···C14^v^	3.5936
C6···H1^xii^	3.3460	H20B···H8^iv^	3.4544
C6···H3^xi^	3.5624	H20B···H13^v^	3.0639
C6···H15^xiii^	3.3733	H20B···H12^v^	3.4957
C7···H8^viii^	3.1161	H20B···H11^v^	3.4741
C7···H15^xiii^	3.1604	H20C···C3^i^	3.5201
C7···H16^xiii^	3.4602	H20C···C4^i^	3.5750
C7···H20B^iv^	3.4084	H20C···C5^i^	3.5450
C7···H12^viii^	3.407 (13)	H20C···C20^xiv^	3.2874
C8···H7^viii^	3.5163	H20C···H1^vi^	3.1705
C8···H8^viii^	2.9850	H20C···H3^i^	3.3696
C8···H16^iv^	3.5808	H20C···H5^i^	3.3902
C8···H18^iii^	3.4145	H20C···H20A^xiv^	3.5351
C8···H19^iii^	3.1087	H20C···H20C^xiv^	2.3622
C8···H20A^iv^	3.1971	H13···O1^v^	3.492 (14)
C8···H20B^iv^	3.2280	H13···C2^x^	3.40 (2)
C9···H18^iii^	2.9091	H13···C3^x^	3.350 (19)
C9···H19^iii^	3.0520	H13···C15^iv^	3.408 (15)
C9···H20A^iv^	2.8631	H13···H2^x^	2.8988
C9···H20B^iv^	3.4819	H13···H3^x^	2.8004
C10···H6^vii^	3.3944	H13···H15^iv^	3.5238
C10···H18^iii^	3.2988	H13···H20B^v^	3.0639
C10···H19^iii^	2.8908	H12···N1^iii^	2.99 (2)
C10···H20A^iv^	3.0175	H12···C7^viii^	3.407 (13)
C11···H2^x^	3.5870	H12···C12^iii^	3.546 (18)
C11···H7^viii^	3.2841	H12···H6^viii^	3.1520
C11···H16^iv^	3.3910	H12···H7^vii^	3.1315
C11···H20A^iv^	3.4725	H12···H7^viii^	2.6565
C11···H20B^v^	3.4783	H12···H20B^v^	3.4957
C12···H7^viii^	3.0068	H12···H12^iii^	2.80 (3)
C12···H20B^v^	3.0819	H11···C2^x^	3.002 (19)
C12···H12^iii^	3.546 (18)	H11···C3^x^	3.518 (17)
C13···H15^iv^	3.3659	H11···H2^x^	2.6953
C13···H20B^v^	2.9736	H11···H3^x^	3.5828
C14···H16^v^	3.5385	H11···H16^iv^	3.4288
C14···H20B^v^	3.5936	H11···H20A^iv^	3.0661
C15···H13^iv^	3.408 (15)	H11···H20B^v^	3.4741
			
C17—O1—C20	118.68 (17)	C1—C2—H2	119.427
C10—N1—C11	118.58 (16)	C3—C2—H2	119.432
C2—C1—C10	120.77 (14)	C2—C3—H3	119.786
C1—C2—C3	121.14 (14)	C4—C3—H3	119.786
C2—C3—C4	120.43 (16)	C4—C5—H5	119.513
C3—C4—C5	122.40 (15)	C6—C5—H5	119.515
C3—C4—C9	119.11 (14)	C5—C6—H6	119.772
C5—C4—C9	118.48 (13)	C7—C6—H6	119.774
C4—C5—C6	120.97 (16)	C6—C7—H7	119.763
C5—C6—C7	120.45 (15)	C8—C7—H7	119.761
C6—C7—C8	120.48 (14)	C7—C8—H8	119.545
C7—C8—C9	120.91 (15)	C9—C8—H8	119.541
C4—C9—C8	118.70 (13)	N1—C11—H11	119.7 (8)
C4—C9—C10	119.14 (12)	C12—C11—H11	117.7 (8)
C8—C9—C10	122.16 (13)	C11—C12—H12	118.1 (8)
N1—C10—C1	122.72 (13)	C13—C12—H12	119.4 (8)
N1—C10—C9	117.83 (11)	C12—C13—H13	116.7 (9)
C1—C10—C9	119.36 (14)	C14—C13—H13	115.5 (9)
N1—C11—C12	122.63 (18)	C14—C15—H15	118.908
C11—C12—C13	122.4 (2)	C16—C15—H15	118.902
C12—C13—C14	127.9 (2)	C15—C16—H16	120.344
C13—C14—C15	120.78 (17)	C17—C16—H16	120.341
C13—C14—C19	122.17 (15)	C17—C18—H18	119.941
C15—C14—C19	117.05 (14)	C19—C18—H18	119.932
C14—C15—C16	122.19 (18)	C14—C19—H19	119.279
C15—C16—C17	119.31 (16)	C18—C19—H19	119.265
O1—C17—C16	125.37 (14)	O1—C20—H20A	109.474
O1—C17—C18	114.76 (17)	O1—C20—H20B	109.470
C16—C17—C18	119.87 (15)	O1—C20—H20C	109.474
C17—C18—C19	120.13 (19)	H20A—C20—H20B	109.465
C14—C19—C18	121.46 (16)	H20A—C20—H20C	109.473
C2—C1—H1	119.613	H20B—C20—H20C	109.471
C10—C1—H1	119.613		
			
C20—O1—C17—C16	−7.9 (2)	C7—C8—C9—C4	−0.0 (3)
C20—O1—C17—C18	173.05 (12)	C7—C8—C9—C10	−179.05 (16)
C10—N1—C11—C12	179.54 (12)	C4—C9—C10—N1	179.14 (14)
C11—N1—C10—C1	−57.2 (2)	C4—C9—C10—C1	2.4 (3)
C11—N1—C10—C9	126.13 (15)	C8—C9—C10—N1	−1.8 (3)
C2—C1—C10—N1	−177.42 (17)	C8—C9—C10—C1	−178.62 (15)
C2—C1—C10—C9	−0.8 (3)	N1—C11—C12—C13	168.88 (13)
C10—C1—C2—C3	−1.0 (3)	C11—C12—C13—C14	−176.86 (12)
C1—C2—C3—C4	1.2 (3)	C12—C13—C14—C15	175.95 (13)
C2—C3—C4—C5	179.28 (17)	C12—C13—C14—C19	−4.6 (3)
C2—C3—C4—C9	0.4 (3)	C13—C14—C15—C16	179.68 (11)
C3—C4—C5—C6	−178.93 (17)	C13—C14—C19—C18	−179.34 (11)
C3—C4—C9—C8	178.80 (15)	C15—C14—C19—C18	0.17 (18)
C3—C4—C9—C10	−2.1 (3)	C19—C14—C15—C16	0.16 (18)
C5—C4—C9—C8	−0.1 (3)	C14—C15—C16—C17	−0.3 (2)
C5—C4—C9—C10	178.92 (15)	C15—C16—C17—O1	−178.93 (12)
C9—C4—C5—C6	−0.0 (3)	C15—C16—C17—C18	0.10 (19)
C4—C5—C6—C7	0.4 (4)	O1—C17—C18—C19	179.35 (11)
C5—C6—C7—C8	−0.5 (4)	C16—C17—C18—C19	0.2 (2)
C6—C7—C8—C9	0.3 (3)	C17—C18—C19—C14	−0.4 (2)

Symmetry codes: (i) *x*+1, *y*−1, *z*+1; (ii) *x*−1, *y*+1, *z*−1; (iii) −*x*+1, −*y*+1, −*z*+1; (iv) −*x*+1, −*y*, −*z*+1; (v) −*x*+2, −*y*, −*z*+1; (vi) *x*, *y*−1, *z*+1; (vii) *x*+1, *y*, *z*; (viii) −*x*, −*y*+1, −*z*+1; (ix) *x*, *y*+1, *z*−1; (x) −*x*+1, −*y*+1, −*z*; (xi) −*x*, −*y*+2, −*z*; (xii) *x*−1, *y*, *z*; (xiii) *x*−1, *y*+1, *z*; (xiv) −*x*+2, −*y*−1, −*z*+2; (xv) *x*+1, *y*−1, *z*.
