# Supplementary figures and images for: Crystal structure of (E)-N-[(E)-3-(4-meth­oxy­phen­yl)allyl­idene]naphthalen-1-amine
Source: Acta Crystallogr Sect E Struct Rep Online. 2014 Oct 24;70(Pt 11):o1174. doi: 10.1107/S1600536814022521 (PMC4257298; doi:10.1107/S1600536814022521)

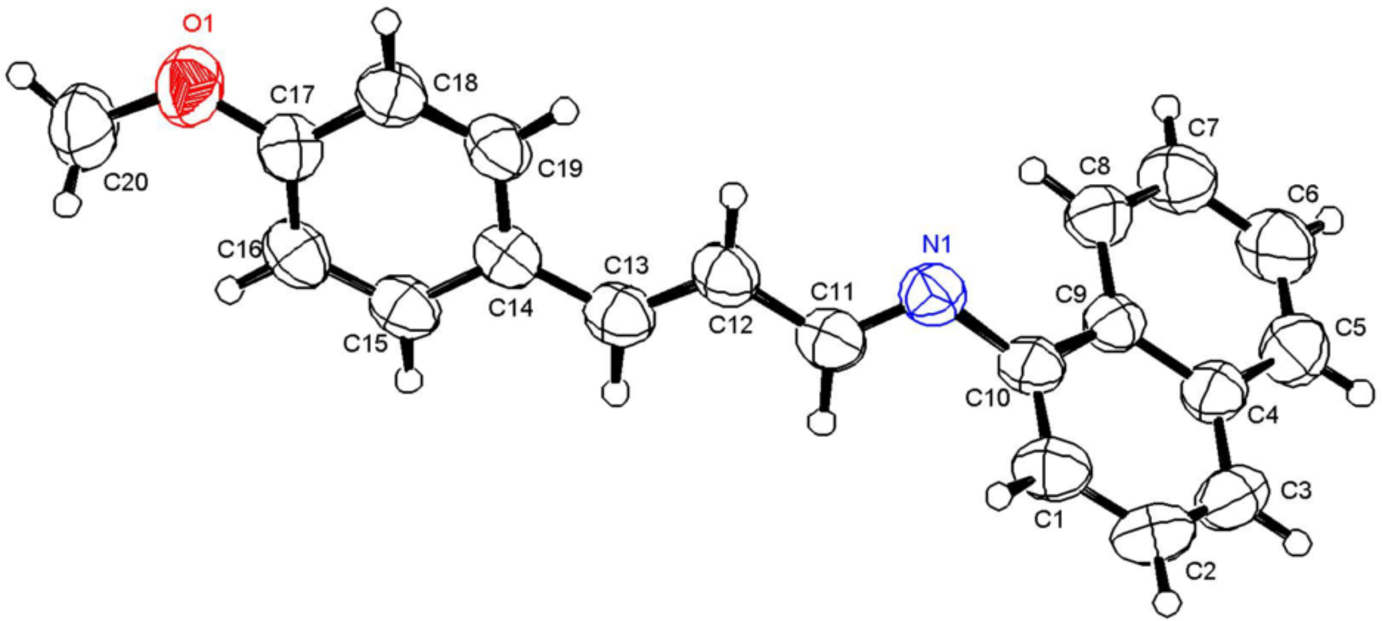

Supplement: Supplementary file 4 [file e-70-o1174-fig1.tif]
